# Antimicrobial Treatment Strategies for *Stenotrophomonas maltophilia*: A Focus on Novel Therapies

**DOI:** 10.3390/antibiotics10101226

**Published:** 2021-10-09

**Authors:** Jean Gibb, Darren W. Wong

**Affiliations:** Division of Infectious Diseases, Keck School of Medicine, University of Southern California (USC), Los Angeles, CA 90033, USA; jean.gibb@med.usc.edu

**Keywords:** *Stenotrophomonas*, *Stenotrophomonas maltophilia*, treatment, cefiderocol, avibactam, aztreonam

## Abstract

*Stenotrophomonas maltophilia* is an urgent global threat due to its increasing incidence and intrinsic antibiotic resistance. Antibiotic development has focused on carbapenem-resistant Enterobacteriaceae, Pseudomonas, and Acinetobacter, with approved antibiotics in recent years having limited activity for Stenotrophomonas. Accordingly, novel treatment strategies for Stenotrophomonas are desperately needed. We conducted a systemic literature review and offer recommendations based on current evidence for a treatment strategy of Stenotrophomonas infection.

## 1. Introduction

*Stenotrophomonas maltophilia* is widely considered an opportunistic, environmental, lower virulence pathogen with a predilection for infection in immunocompromised patients. However, while community-acquired acquisition does occur [1], Stenotrophomonas predominantly occurs as a nosocomial infection, most commonly pneumonia, but also causing bloodstream, intra-abdominal, urinary, catheter and implanted device-associated, and more rarely, endocardial, bone, soft tissue, and nervous system infections [2]. Despite its perception as a low virulence pathogen, the crude mortality rate for patients with Stenotrophomonas bacteremia has ranged from 14 to 69% [2,3,4]. Additionally, infections caused by Stenotrophomonas have an increasing worldwide prevalence and are now reported as the most common carbapenem-resistant Gram-negative bloodstream pathogen and sixth most common cause of intensive care pneumonia in the United States [2,5,6,7]. Infections due to Stenotrophomonas are especially significant due to intrinsic antibiotic resistance, often resulting in either delayed initiation of active antimicrobial therapy or the necessity for higher toxicity or less efficacious antibiotic agents. Consequently, meta-analyses highlight a significantly higher mortality in patients treated with an initially inappropriate antibiotic, 61% as opposed to 30% [3]. Antibiotic resistance is mediated by the combination of plasmid-mediated resistance, integrons, insertion sequence common region elements, antibiotic modification enzymes, multidrug efflux pumps, and reduced outer membrane drug permeability [2,7,8,9]. However, most significant is the presence of two intrinsic chromosomal inducible β-lactamases, L1 and L2, which encode an Ambler class B metallo-β-lactamase and an Ambler class A serine-cephalosporinase, cumulatively conferring expansive resistance to β-lactam and carbapenem therapies [2]. These attributes make infection caused by *Stenotrophomonas maltophilia* a particular challenge with a dire need for additional treatment options. Our review seeks to highlight the evidence supporting current treatment as well as review novel therapeutics in order to provide a recommended strategy for management.

## 2. Methods

We conducted a systematic review of the literature to examine Stenotrophomonas therapeutics. A search was performed to identify any published literature regarding in vitro analysis of therapies as well as clinical management of Stenotrophomonas with a specific focus on recent literature, novel therapeutics, and resistant Stenotrophomonas—particularly cases involving alternatives to trimethoprim-sulfamethoxazole. Search parameters were kept broad to maximize capture of relevant references. Searches for “Stenotrophomonas”, “resistance”, AND “antibiotic” using the PubMed search engine and secondary search parameters of “Stenotrophomonas” AND “treatment” were also performed. The literature search was performed on 5/17/21. References from identified articles were also reviewed to identify other relevant studies.

## 3. Results

A total of 1493 manuscripts were identified following the PubMed search. These results were reviewed to identify relevant studies focusing on antimicrobial treatment of Stenotrophomonas. Manuscripts which focused on virulence factors or epidemiologic factors were excluded as they were deemed not to be the focus of our review. Additionally, single case reports were excluded unless they described successful management with a novel treatment regimen. There were 52 articles found to be relevant to our analysis of Stenotrophomonas therapeutics, which were used to identify additional relevant studies.

### 3.1. Historical Therapeutics

#### 3.1.1. Trimethoprim-Sulfamethoxazole (TMP-SMX)

The mainstay of treatment for *Stenotrophomonas* infections is trimethoprim-sulfamethoxazole (TMP-SMX) and it remains the current drug of choice. However, while sensitivity to TMP-SMX generally remains high, with estimates ranging from 79 to 96%, global resistance has been increasing albeit with significant regional variability, particularly amongst cystic fibrosis patients [2,10,11]. There is a wealth of clinical experience using TMP-SMX to treat serious *S. maltophilia* infections. Treatment success rates assessed by microbiological eradication and clinical cure frequently favor TMP-SMX over other commonly used agents when the organism is reported sensitive in vitro [12,13]. However, despite the historical emphasis on TMP-SMX, there is mounting evidence that levofloxacin and minocycline may have comparable if not equal efficacy rates in specific clinical scenarios [7,14,15]. Optimized TMP-SMX dosing is essential for effective treatment of *S. maltophilia* infection with additional concern for the possibility of underdosing in critically ill patient populations. The generally recommended dose is 15–20 mg/kg/day of the TMP component in divided doses [12,14]. Therapeutic drug monitoring is not routinely available and optimal dosing strategies are not fully defined. In critically ill patients on hemodialysis, evidence suggests an initial 24–48 h of administration at 15 mg/kg/day TMP followed by 7.5 mg/kg after each four-hour hemodialysis session. In patients on continuous veno-venous hemodiafiltration, subtherapeutic dosing is a significant concern and aggressive dosing at 15 mg/kg/day is suggested [16].

#### 3.1.2. Fluoroquinolones

In the setting of TMP-SMX resistance or when administration is contraindicated because of life-threatening allergy or other clinical factors, quinolones are frequently utilized as second-line therapy [17]. In patients who are relatively quinolone-naive, levofloxacin susceptibility is reported in the range of 80–91% [12,13]. Levofloxacin has the highest availability of literature; however, some reports have suggested moxifloxacin in vitro was reported to have the lowest minimum inhibitory concentration (MIC) amongst all quinolones, while both levofloxacin and moxifloxacin were both significantly lower than ciprofloxacin [18,19]. Comparable outcomes have been described in retrospective studies when comparing quinolone monotherapy to TMP-SMX with no differences identified in microbiological cure or 30-day mortality [13,20,21]. However, development of quinolone resistance was described by Cho et. al, finding that in patients initially treated with levofloxacin who developed recurrent bacteremia, 50% of isolates had developed quinolone resistance [20]. The more frequent development of resistance to therapy with levofloxacin in comparison to TMP-SMX suggests that the barrier to resistance in quinolones, frequently mediated via efflux pumps or mutation of the drug target, is lower [12,22]. Nearly 20% of patients with *S. maltophilia* pneumonia who do not achieve microbiological eradication may develop quinolone resistance after levofloxacin exposure as opposed to 7–8% of those exposed to TMP-SMX [12,22]. This is especially relevant in cystic fibrosis or cirrhotic patients for whom frequent or chronic quinolone exposure is common, and for deep-seated infections where the duration of antibiotic therapy is longer than 1–2 weeks.

#### 3.1.3. Minocycline

In the tetracycline family, minocycline susceptibility is reported to be greater than 95% for *S. maltophilia* based on CLSI-defined breakpoints (Clinical and Laboratory Standards Institute) [2]. Consistent with these findings, 1289 *S. maltophilia* specimens were collected between 2014 and 2018, the majority from respiratory specimens, and were found to exhibit in vitro sensitivity to minocycline at 99.5%, which notably remained active in nearly 93% of TMP-SMX non-susceptible strains [23]. Minocycline carries minimal drug–drug interactions and a relatively good tolerability profile [14]. In one retrospective review of 284 patients, the 30-day mortality for patients treated primarily for *S. maltophilia* pneumonia with minocycline vs. TMP-SMX was 5.1% vs. 14.7%, respectively, with an adjusted odds ratio favoring minocycline, though this did not meet statistical significance. Additionally, more frequent adverse events did occur in the TMP-SMX group [14]. Another retrospective study found no difference between treatment with monotherapy minocycline when compared to TMP-SMX; although, minocycline patients were treated for a longer duration (mean 13 vs. 7 days) and TMP-SMX dosing (average 8.5 mg/kg/day) may have introduced confounding [24]. A case of *S. maltophilia* endocarditis was also successfully treated utilizing oral minocycline as part of a combination treatment regimen [25]. The multidrug efflux pumps that convey resistance to TMP-SMX often convey resistance to quinolones and β-lactams/β-lactamase inhibitors contemporaneously but do not appear to affect minocycline susceptibility; minocycline is often utilized in the context of TMP-SMX and levofloxacin resistance [26].

In scenarios where minocycline has been used for treatment, the most common dose is 100 mg twice daily, often with an initial 200 mg loading dose. Current interpretive susceptibility breakpoints of minocycline for Stenotrophomonas are a minimum inhibitory concentration (MIC) ≤ 4.0 μg/mL. Manufacturer pharmacokinetic studies of minocycline reported a mean peak concentration of 4.2 μg/mL after a single 200 mg dose; however, other investigators have reported a range from 3.0 to 6.2 μg/mL in peak concentrations reached, notably with serum levels consistently higher than other tetracyclines or tigecycline [27]. Additionally, minocycline exhibits a large volume of distribution resulting in high tissue penetration, with levels consistently exceeding those in serum, particularly favorable in lung tissue with an estimated tissue:serum concentration of 3.80 [27]. Consequently, Monte Carlo simulation predicted an effective target attainment of approximately 95% for treatment of pneumonia due to susceptible strains of *S. maltophilia*, with a minocycline dosing regimen of 100 mg every 12 h [28]. One additional caveat is the limited urinary excretion of minocycline, with an estimated 5–12% of a dose being recovered in urine [27]. Reports utilizing minocycline for Gram-negative bacteremia have been comparably sparse, in anecdotal and case reports, often as a component of a combination antibiotic regimen [27]. There was decidedly scant literature focusing on oral minocycline; however, its high bioavailability makes transition to oral therapy an attractive option in clinically responsive patients.

At least in one study of 41 diverse strains of Stenotrophomonas, minocycline was comparably if not more active in vitro than the newest classes of tetracyclines, including tigecycline, eravacycline, and omadacycline [29].

#### 3.1.4. Beta-Lactam Treatment

The intrinsically produced β-lactamases L1 and L2 in *S. maltophilia* confer broad-range resistance to β-lactams, cephalosporins, and carbapenems [4,29]. Despite this, ceftazidime is a weak inducer of L2 (a serine β-lactamase with cephalosporinase hydrolysis), and a breakpoint for ceftazidime in clinical settings is provided by the CLSI [30]. Clinical success rates have been reported at 66% with ceftazidime monotherapy for *S. maltophilia* infections in cases with TMP-SMX resistance, with limited datasets [31]. The utility of ticarcillin has largely relied on its coadministration with clavulanate for its inhibition of the L2 serine-β-lactamase; in vitro susceptibility rates range from 18 to 91% [32,33].

#### 3.1.5. Colistin and Polymyxin

Colistin is used as salvage therapy in many multidrug resistant (MDR) Gram-negative infections, but its role is limited by significant nephrotoxicity, the availability of newer, more effective, and less toxic antimicrobials, and poorer outcomes when compared to the newer counterpart agents that are commercially available [34]. Susceptibility testing is hampered by *Stenotrophomonas*’ multifaceted mechanisms of resistance to the drug; resistance mechanisms are frequently adaptive, sometimes harbored by heterogeneous subpopulations, and may only emerge upon drug exposure [35]. Rates of in vitro susceptibility range from 9 to 86% and typically require high concentrations to achieve growth inhibition [11,36,37]. Colistin in combination with other agents is frequently of interest in an effort to address multiple resistance mechanisms [34,38]. In vitro checkerboard assays have demonstrated some success with combination N-acetylcysteine-colistin and telavancin-colistin combination therapy [38,39]. In a series of 24 strains of *S. maltophilia* resistant to TMP-SMX and rifampicin, colistin combination therapy with both agents showed synergy in 60% of isolates [34]. Similarly, the combination of colistin and rifampin was the only reliably bactericidal combination in both in vitro and a subsequent simple invertebrate experimental model [40].

#### 3.1.6. Combination Treatment

Combination therapy garners interest as *S. maltophilia* phenotypes with accumulated resistance mechanisms become increasingly widespread. Clinical data demonstrating superior outcomes are lacking. As previously described, checkerboard in vitro assays may demonstrate in vitro synergy and restored susceptibility to otherwise resistant isolates but translation to clinical outcomes has not been demonstrated [26,34,39,41,42]. Experience with combination therapy is limited to isolated case reports describing clinical success [43,44]. In a review of 43 cases of *S. maltophilia* endocarditis, the majority were treated with combinational antibiotic regimens, albeit overall mortality reached 35% [45]. In contrast, a retrospective review of pneumonia with monotherapy compared to combination therapy in permutations with TMP-SMX, levofloxacin, minocycline, and ceftazidime found no discernible advantage emerged for the combined approach and the development of resistance to the utilized agents was not reduced [46]. Another study found that while in vitro synergy was described, a retrospective review of a small series of 29 cancer patients with Stenotrophomonas bacteremia did not find an improved prognosis with combination therapy compared to monotherapy [47]. Similarly, a retrospective cohort study of adult patients with Stenotrophomonas pneumonia compared 61 who were treated with monotherapy compared to 45 who received combination treatment. Patients in the combination subgroup had higher disease severity by Charlson Comorbidity Index and APACHE II score; however, accounting for this on multivariate analysis, there was no discernable difference in mortality, clinical response, microbiological eradication, or resistance development between both treatment groups [48].

### 3.2. Novel Therapeutics

There has been considerable interest in novel treatment options for Stenotrophomonas. Experimental antibiotics with either limited or negligible in vitro activity for Stenotrophomonas include the novel oral carbapenem, CS-834 [49]. Similarly, none of the novel β-lactamase inhibitor combinations have reliable Ambler class B β-lactamase inhibition; thus, relebactam-imipenem and vaborbactam-meropenem were not found to exhibit Stenotrophomonas activity [50]. The data on ceftazidime-avibactam are more equivocal; Moriceau et. al and Lin et al. describe decreases in minimum inhibitory concentration (MIC) with ceftazidime-avibactam compared to ceftazidime alone [51,52]. However, a significant subset of isolates remained resistant to the combination of ceftazidime-avibactam and additional studies reported only a minimal improvement to ceftazidime with the addition of avibactam [53]. Ceftolozane-tazobactam in vitro data for Stenotrophomonas are very limited, with Gramegna et al. and Farrell et. al describing a predicted in vitro sensitivity rate of 60 and 40%, respectively [54,55]. Conversely, Grohs et al. reported 12/12 Stenotrophomonas isolates from cystic fibrosis patients were resistant to ceftolozane-tazobactam [56]. Plazomicin, similar to all other aminoglycosides, demonstrated poor activity for Stenotrophomonas [57].

#### 3.2.1. Eravacycline and Tigecycline

Tigecycline in vitro MICs are reported favorable, although no CLSI-defined breakpoints are available [17,58]. Using breakpoints extrapolated for Enterobacteriaceae, 85–90% of S. maltophilia isolates are susceptible in vitro with an MIC < 2 mg/L [15]. Small case series reporting its use in treatment of *Stenotrophomonas* ventilator-associated pneumonia in trauma patients have had clinical success with variable success in microbiological cure [59]. Comparison of tigecycline to TMP-SMX for treatment of *S. maltophilia infection* in one retrospective series produced comparable clinical outcomes and higher microbiological cure in the tigecycline group, though authors noted no patients with primary bacteremia were treated with tigecycline out of concern for suboptimal serum drug levels [60]. In contrast, a retrospective cohort of 82 patients in three tertiary Chinese hospitals with ventilator-associated pneumonia was reviewed: 46 who received tigecycline and 36 fluroquinolone therapy. In comparison with fluoroquinolone treatment, patients treated with tigecycline were found to have a significantly worse clinical cure (32.6% compared to 63.9%) and a lower rate of microbiological cure (28.6% as opposed to 59.1%) with a trend towards increased 28-day mortality (48% vs. 28%), although this did not reach statistical significance [61]. The authors postulated subtherapeutic tigecycline drug levels at standard dosing as one potential contributor to these findings. Indeed, one concern of tigecycline is that current susceptibility breakpoints are insufficient with mean reported peak concentrations following 50 mg and 100 mg dose of tigecycline at 0.38 mg/L and 0.91 mg/L, respectively [62]. There is also significant discrepancy in achievable drug levels in the lung, with reported data from epithelial lining fluid studies in ventilated patients being concerning for insufficient levels [62]. Monte Carlo simulation predicted that with standard dosing of tigecycline of 50 mg every 12 h, predicted target attainment was actually >99% for strains with an MIC ≤ 0.5 µg/mL. However, predicted target attainment decreased significantly with increasing MIC at 71 and 11% at MIC of 1 and 2 µg/mL, respectively [28]. Therefore, a prospective study of critically ill intensive care patients evaluated tigecycline concentration in lung tissue. Patients were treated with high-dose tigecycline (100 mg every 12 h following 200 mg loading dose) and achieved target steady state epithelial lining fluid levels predictive of clinical success in 95% of pneumonia cases for a pathogen MIC < 0.25 µg/mL, albeit predicted success rate decreased to 41–75% if pathogen MIC was 0.5–1 µg/mL [63]. Accordingly, interest in higher dosing regimens of tigecycline has been proposed, with at least one case reporting successful utilization of high-dose tigecycline for Stenotrophomonas bacteremia [64]. Eravacycline is increasingly utilized in MDR Gram-negative infections but has limited data to reflect its use in treatment of *Stenotrophomonas* infections [65]. In several series, the reported MICs for eravacycline are 2- to 4-fold lower than for tigecycline; however, in a series of 118 isolates, Zhanel et al. described identical MICs for eravacycline in comparison to tigecycline [50,66]. It is worth noting that eravacycline MICs frequently mirror minocycline for *S. maltophilia* [66]. In another larger collection of over 1000 isolates, 90% were inhibited by eravacycline at an MIC of 2 ug/mL. Some reports note fewer gastrointestinal side effects with eravacycline in comparison to tigecycline [65].

#### 3.2.2. Cefiderocol

Cefiderocol’s novel mechanism involves the use of a siderophore for active transport into the bacterium, conjugated with a cephalosporin that closely resembles cefepime and ceftazidime [67]. It is of great interest in the treatment of MDR Gram-negative infections due to its stability against both serine and metallo-β-lactamases. In *S. maltophilia* isolates, MIC may exhibit a wide range but MIC_90_s have been reported as consistently favorable [5,67,68]. Retained susceptibility has been demonstrated in isolates resistant to TMP-SMX, levofloxacin, and polymyxin [5]. However, to date, clinical experience is extremely limited and reliant on experimental and animal models. A randomized phase 2 double-blind trial reported non-inferiority between cefiderocol and imipenem-cilastatin in the treatment of patients with suspected resistant Gram-negative urinary infections; however, there were no cases of Stenotrophomonas in either study group [69]. The CREDIBLE-CR trial was a randomized open-label multicenter study comparing cefiderocol to best available therapy. Only five cases of *S. maltophilia* infection were enrolled: all pneumonia cases and all in the cefiderocol treatment group. In this small sample size, cefidercol MICs were favorably low; however, the treatment response for all five cases was deemed indeterminate and all-cause mortality was 80% (4 of 5) at the end of study [70]. Conversely, in vivo murine thigh and lung models of Stenotrophomonas infection showed highly effective response to cefiderocol [71,72]. Similarly, in a series of MDR bloodstream isolates, cefiderocol’s in vitro MICs were definitively favorable in comparison to imipenem-relebactam, ceftazidime-avibactam, and ceftolozane-tazobactam [73]. This finding was also reproducible in a report of 127 Stenotrophomonas clinical isolates from Italy, where cefiderocol exhibited 100% susceptibility. This compared favorably to colistin, ceftazidime-avibactam, and ceftolozane-tazobactam, which exhibited susceptibility rates of 69, 41, and 37%, respectively [74]. However, it is important to highlight that there are no MIC breakpoints provided by CLSI for cefiderocol interpretation in *S. maltophilia* and its role is primarily in MDR and extensively drug resistant (XDR) infections [67]. When resistance in *S. maltophilia* occurs, the mechanism is not defined [67].

#### 3.2.3. Avibactam-Aztreonam

The approach to *S. maltophilia* infections with combination aztreonam and avibactam therapy continues to gain interest due to the potential capacity for evasion of both chromosomally encoded L1 and L2 β-lactamases [17,51]. Avibactam has attracted attention for its ability to covalently bind to L2 and thereby inhibit its activity [4,75,76]. Unfortunately, in experimental models, ceftazidime-avibactam resistant mutants showed a compensatory hyperproduction of L1 reducing the effectiveness ceftazidime in this scenario [76]. Additionally, avibactam is deactivated by L1 at a low rate, which may compromise its utility in hyperproducing L1 strains [76]. Although none of the commercially available inhibitors bind to the L1 metallo-β-lactamase, aztreonam is unique in that it is not a substrate [4,17]. Therefore, the combination of aztreonam and avibactam hinges on the inhibition of the L2 serine β-lactamase by avibactam in order to allow aztreonam to evade the L1 metallo-β-lactamase and exert a bactericidal effect [4,17]. This has been demonstrated in vitro with *Stenotrophomonas* isolates resistant to aztreonam that have re-gained aztreonam susceptibility with the addition of avibactam [30,51,77].

In a case series of 12 isolates, the addition of avibactam to aztreonam reduced the in vitro susceptibility to aztreonam in *S. maltophilia* significantly [30,51,77]. This approach has been used successfully in limited case reports. The combination of aztreonam-avibactam was effective in treatment of a case of bacteremia in a renal transplant recipient with an XDR *S. maltophilia* isolate exhibiting in vitro resistance to TMP-SMX, minocycline, and levofloxacin [75]. A second case reported successful treatment of tricuspid valve endocarditis with septic emboli due to pan-drug-resistant *S. maltophilia* treated with ceftazidime-avibactam and aztreonam in combination with surgical therapy [78].

At present, a combined formulation of avibactam and aztreonam is not widely available. Therefore, in salvage situations, the combination of ceftazidime-avibactam and aztreonam has been used. However, an optimal dosing strategy has not been established. The largest reported cohort of 102 cases utilized a regimen of ceftazidime/avibactam 2.5 gm every 8 h administered as an extended 8 h infusion in half of patients and aztreonam 2 gm every 8 h as a 2 h infusion [79]. A hollow fiber infection model examining different dosing regimens suggested a preferred regimen of simultaneous administration of ceftazidime/avibactam with aztreonam as opposed to staggered sequential administration, no difference between extended infusion and or standard infusion rate, and possible superiority of 8gm of daily aztreonam. This modelling was based on two New Delhi metallo-β-lactamase Enterobacteriaceae strains [80,81].

#### 3.2.4. Phage and Peptide Therapy

Bacterial phage therapy is gaining traction as a novel approach to refractory cases of extremely multidrug resistant (XDR) bacterial infections; advantages include specificity to the target, limited side effects on the host biome, and lack of major end organ damage [82]. In 2004, Chang et al. reported a series of *Stenotrophomonas* bacteriophages, ϕSMA1–ϕSMA8, with an in-depth characterization and discussion of one phage, ϕSMA5 [83]. In this series, they describe the exquisite specificity of phages to their hosts and their bactericidal effect [83]. Several phages have since been developed against *S. maltophilia* utilizing virulence factors as targets; as recently as 2020, a novel phage targeting the pilus on the surface of *S. maltophilia* was developed by McCutcheon et al., with promising results [82].

Peptide therapy is another novel approach garnering interest for the treatment of XDR infections. Interest stems primarily from the role of naturally occurring antimicrobial peptides in the coordination of the innate immune system [84]. In a review of three alpha helical peptides and their in vitro activity against *S. maltophilia* isolates from cystic fibrosis patients, minimum inhibitory concentrations (MICs) were markedly lower than for tobramycin under multiple experimental conditions [84].

### 3.3. A Recommended Approach to Therapy

Based on the best available evidence, we postulate a preferred treatment strategy for *Stenotrophomonas maltophilia* infection. The best scenario would be to reduce the likelihood of infection entirely through antibiotic stewardship and judicious antibiotic use, particularly of carbapenems, which have been consistently identified in several studies as a multivariate risk factor for Stenotrophomonas acquisition [2]. Additionally, cornerstone source control principles with early drainage of abscesses and a strict requirement for removal of catheters and foreign body material should be a priority due to the predisposition of Stenotrophomonas for biofilm production [2]. Based on overall low reported rates of sensitivity, historically low clinical success rates, and the high plausibility of inducible resistance, we do not recommend routine ceftazidime monotherapy or ticarcillin therapy, even if in vitro sensitivity is predicted. The development of resistance by Stenotrophomonas has been well described and therefore, combination treatment strategies have been proposed to increase treatment efficacy and reduce resistance induction. While in vitro studies with combination regimens appear promising, clinical data are lacking and, based on current evidence, combination regimens have not shown a benefit in either clinical response or prevention of resistance development. Therefore, for routine disease conditions, pneumonia, catheter-associated bacteremia, urinary tract infection, or bacteremia, we do not advocate the routine use of combination treatment. However, in cases when source control is not achievable, such as in abdominal perforation with undrained abscess, a plausible case could be made to utilize two active agent therapies. Additionally, management of Stenotrophomonas endocarditis, an overall rare entity, has historically been treated with combination therapy and the authors felt there was a lack of sufficient evidence in the literature to recommend against this approach. Additionally, when polymyxin or colistin, as less preferred treatment agents, are required, then the use of combination therapy may be a viable option based on patient clinical severity.

Consistent with historical evidence, we recommend that trimethoprim/sulfamethoxazole, with aggressive dosing of at least 15–20 mg/kg/day, be the preferred treatment in all scenarios due to its high in vitro susceptibility and reported efficacy. However, when toxicity or drug allergies preclude its use, then sensitivities permitting, fluoroquinolones, with levofloxacin the most studied, and moxifloxacin, a preferred secondary alternative, can be an option. Levofloxacin dose should be at least 500 mg daily, although 750 mg daily is preferred provided renal clearance permitting. Fluoroquinolones have shown comparable clinical responses when compared to TMP-SMX, in particular, for the treatment of pneumonia or bacteremia with appropriate early source control. The caveat is that we do not recommend fluoroquinolones for deep-seated abscesses, endovascular infection, or disease processes that require prolonged antibiotic therapy due to the low resistance barrier and well described recovery of resistant isolates after even relatively brief durations of therapy. In scenarios where neither fluoroquinolone nor TMP-SMX are an option due to either in vitro resistance, toxicity, or incompatible disease process, several options can be considered. When comparing minocycline, tigecycline, and eravacycline, susceptibility to this class appears to be retained in the presence of TMP-SMX and quinolone resistance. In vitro MICs may favor minocycline and eravacycline over tigecycline but the in vitro sensitivity for all three is favorable with MIC_90_ < 2 ug/mL for > 90% of *S. maltophilia* isolates. Therefore, we recommend minocycline as the preferred option unless polymicrobial infection is present, which may necessitate either tigecycline or eravacycline for consolidated therapy. Pharmacokinetic studies may in fact support minocycline with high predicted target attainment with a dosing regimen of 200 mg load followed by 100 mg every 12 h. Treatment of primary bacteremia or urinary infections with minocycline, tigecycline, or eravacycline should proceed with caution when other agents with more optimized serum levels are available and, in these circumstances, higher dosing regimens may be preferrable. Additionally, while the susceptibility breakpoint for tigecycline is <2 µg/mL, there remains a concern regarding possible heightened clinical failure due to subtherapeutic drug levels. In *Stenotrophomonas maltophilia* isolates with an MIC < 0.5 µg/mL, standard dosing of tigecycline 50 mg every 12 h following a 100 mg loading dose can be used; however, in cases with increased MIC, particularly those at ≥0.5–1 µg/mL, a higher dosing regimen of 200 mg loading dose followed by 100 mg every 12 h is recommended. Of specific mention is that increasing evidence has supported the non-inferiority of oral antibiotics compared to intravenous administration. Therefore, as fluoroquinolones, minocycline, and TMP-SMX all exhibit high bioavailability, it is justifiable that these agents could be provided by oral administration.

In case of XDR *Stenotrophomonas maltophilia* infection exhibiting resistance to the preferred first-line treatment options, the two most promising options appear to be the combination of aztreonam-avibactam or cefiderocol. However, both treatment options do have limitations. Polymyxins do have a role as historical salvage therapy with the caveat that polymyxin therapy is associated with less effective clinical response and often prohibitive drug toxicity. Therefore, in septic shock conditions, colistin remains a potentially viable line option for initial therapy. In the case of pneumonia, administration of both intravenous and inhaled colistin would be recommended. There is some in vitro literature postulating a benefit with the addition of either N-acetylcysteine or rifampin; however, clinical data are lacking and thus, it was difficult to recommend routine use of either as adjunctive therapy. However, if colistin is used, we recommend this be for a short duration pending additional susceptibility testing to allow for transition to an alternative therapeutic option as soon as possible. Phage therapy has been used with limited success for other resistant bacteria, but no reports exist for clinical application with Stenotrophomonas. Thus, phage options may be a consideration in the setting of chronic soft tissue infection but is unlikely to be available for implementation in an acute clinical setting.

In cases of MDR bloodstream isolates, there has not been comparison between aztreonam-avibactam and cefiderocol for salvage therapy. Cefiderocol has favorable reported in vitro MIC data when compared to currently available β-lactam–β-lactamase inhibitor combinations. However, there is a paucity of clinical data to reflect its use. In the CREDIBLE-CR trial, all Stenotrophomonas cases exhibited indeterminate response to therapy with an 80% mortality, albeit with only 5 cases. In clinical practice, sensitivity testing and interpretable breakpoints do not currently exist for cefiderocol and *S. maltophilia*. Additionally, the mechanism of resistance to cefiderocol is unknown; therefore, development of resistance while on therapy may occur. The combination aztreonam-avibactam has a plausible mechanism of action and at least sparse case reports of clinical efficacy. Microbiology laboratory testing for aztreonam-avibactam susceptibility is not likely to be readily available, which further complicates this regimen. Therefore, we consider both options of cefiderocol and aztreonam-avibactam comparable based on very limited data. Importantly, co-formulation of aztreonam-avibactam is not available; administration of ceftazidime-avibactam and aztreonam is recommended for salvage therapy. Based on the available literature, ceftazidime-avibactam 2.5 gm every 8 h with simultaneous infusion of aztreonam 2 gm every 8 h is recommended and in patients with decompensated shock, a justifiable case could be made to utilize 2 gm every 6 h. Accordingly, selection between both salvage options should be based on drug availability, drug toxicity considerations, and the requirement for concurrent activity of co-pathogens.

The above strategies are highlighted in Table 1 and offer a framework for the treatment of Stenotrophomonas infection. Ultimately, *Stenotrophomonas maltophilia* will continue to be a challenging pathogen with evolving strategies needed, particularly with widespread utilization of more and more broad spectrum Gram-negative antimicrobials that will result in an increasing frequency of Stenotrophomonas recovery in nosocomial settings.

## Figures and Tables

**Table 1:** Recommended treatment approach for Stenotrophomonas infection.

Agent	Line of Treatment	Notes on Clinical Use
Trimethoprim-Sulfamethoxazole (TMP-SMX)	First line	Increasing resistance amongst cystic fibrosis patients Optimized dosing essential (15–20 mg/kg TMP-component in divided doses every 6–8 h)
Fluoroquinolones	First line	Low resistance barrier Caution in deep-seated infections or anticipated duration of use >14 days Levofloxacin most studied—recommended dose 750 mg every 24 h, moxifloxacin a viable alternative
Tetracyclines	First line	Favorable tolerability profile and large volume of distribution to tissues Susceptibility generally retained in the context of TMP-SMX and/or levofloxacin resistance Minocycline preferred agent—200 mg loading dose, followed by 100 mg every 12 h Susceptibility of tigecycline and eravacycline mirror minocycline; tigecycline 100mg loading dose then 50 mg every 12 h; however, if *S. maltophilia* MIC > 0.5 µg/mL, then recommend 200 mg loading dose, then 100 mg every 12 h Low drug recovery from urine and lower serum levels should raise caution with treatment for bacteremia and urinary infections; utilization of high dosing regimens may be preferred in these circumstances
Cefiderocol	Salvage	Favorable reported MIC (minimum inhibitory concentration) data Limited to experimental and animal models Utility primarily in XDR ^1^ isolates
Ceftazidime/Avibactam + Aztreonam	Salvage	Efficacy is largely in vitro Clinical evidence limited to case reports Susceptibility testing for combination therapy not routinely available Ceftazidime/Avibactam 2.5 gm every 8 h CONCURRENT Aztreonam 2 gm every 8 h (8 gm daily if septic shock) Used in XDR ^1^ isolates
Combination therapy amongst first/second line agents and alternatives	Possible options	Lack of evidence to support routine clinical benefit or reduction in emergence of resistance Success limited to case reports/series Can be considered in deep seated/polymicrobial infections; still standard of care in endocarditis/endovascular disease
Polymyxins	Possible options	Susceptibility testing difficult to perform and concern for heterogenous resistance Use limited by toxicity and reported lower efficacy compared to preferred options Can be used as empiric therapy pending alternative options When used for pneumonia-intravenous and nebulized therapy recommended

^1^ Extensively drug-resistant Stenotrophomonas species, defined by resistance by CLSI breakpoints to TMP-SMX, levofloxacin, and minocycline.
